# Multiple intra-articular injections with adipose-derived stem cells for knee osteoarthritis cause severe arthritis with anti-histone H2B antibody production

**DOI:** 10.1016/j.reth.2023.06.007

**Published:** 2023-06-26

**Authors:** Y. Hosono, A. Kuwasawa, E. Toyoda, K. Nihei, S. Sato, M. Watanabe, M. Sato

**Affiliations:** a Division of Rheumatology, Department of Internal Medicine, Tokai University School of Medicine, 143 Shimokasuya Isehara, Kanagawa, 259-1193 Japan; b Saitama Cooperative Hospital, 1371 Kisoro, Kawaguchi, Saitama, 333-0831, Japan; c Department of Orthopaedic Surgery, Surgical Science, Tokai University School of Medicine, 143 Shimokasuya, Isehara, Kanagawa, 259-1193 Japan; d Center for Musculoskeletal Innovative Research and Advancement (C-MiRA), Tokai University Graduate School, 143 Shimokasuya, Isehara, Kanagawa, 259-1193 Japan

**Keywords:** Adipose-derived stem/stromal cells, Anti-histone H2B antibody, Osteoarthritis, Knee joint

## Abstract

**Introduction:**

Osteoarthritis (OA) is the most common form of arthritis. OA results from the breakdown of cartilage, which leads to deterioration of the entire joint and the connective tissue that holds the joint together, and gradually and irreversibly worsens over time. Adipose-derived stem/stromal cells (ADSCs) have been used in the treatment of knee OA. However, the safety and efficacy of ADSC treatment of OA remain unclear. In this study, we investigated the pathophysiology of severe knee arthritis that occurred after ADSC treatment by screening for autoantibodies in synovial fluid from patients who received ADSC treatment.

**Methods:**

Adult Japanese patients with OA who received ADSC treatment at Saitama Cooperative Hospital between June 2018 and October 2021 were enrolled. Antibodies (Abs) were screened using immunoprecipitation (IPP) with [^35^S]-methionine-labeled HeLa cell extracts. The detected protein was identified by liquid chromatography coupled with time-of-flight mass spectrometry (MS) and ion trap MS, and the corresponding proteins were confirmed as autoantigens using immunoblotting. Ab titers were measured using an enzyme-linked immunosorbent assay.

**Results:**

A total of 113 patients received ADSC treatment, and 75% (85/113) received ADSC injection at least twice with a 6-month interval between. No obvious abnormalities were observed in any patient after their first treatment; by contrast, 53% (45/85) of patients who received their second or third ADSC injection showed severe knee arthritis. IPP detected a common anti-15 kDa Ab in synovial fluid of 62% (8/13) of the samples analyzed from patients who showed severe arthritis. This Ab was not detected in synovial fluid obtained from the same joints before treatment. The corresponding autoantigen was identified as histone H2B. All available synovial samples from patients who tested positive for anti-histone H2B Ab were newly positive after the treatment; that is, none had been positive for anti-histone H2B Ab before treatment.

**Conclusions:**

Multiple ADSC injections for OA induced severe arthritis in a high percentage of patients, particularly after the second injection. Synovial fluid from some patients with knee arthritis contained Ab to histone H2B that appeared only after ADSC treatment. These findings provide new insights into the pathogenesis of ADSC treatment-induced severe arthritis.

## Highlights

• Multiple injections of ADSCs into knees with OA induced severe arthritis.
• Synovial fluid of patients with severe arthritis contained anti-histone H2B antibody.
• One highlight added as follows Anti-histone H2B antibody production (in synovial fluid) was induced by multiple ADSCs injections.

## Abbreviations

ADSCs: Adipose-derived stem/stromal cells
Abs: Antibodies
DILE: drug-induced lupus erythematosus
ELISA: Enzyme-linked immunosorbent assay
HHDNA: human histone H2A-H2B-DNA
IPP: immunoprecipitation
LCMS-IT-TOF: a liquid chromatograph mass spectrometer that combines a high-performance liquid chromatograph with an ion-trap (IT) mass spectrometer and time-of-flight (TOF) mass spectrometer
MS: mass spectrometry
MSCs: multipotent stem cells
MS/MS: Tandem mass spectrometry
OA: Osteoarthritis (OA)
PAGE: polyacrylamide gel electrophoresis
PVDF: polyvinylidene fluoride
SDS: sodium dodecyl sulfate
SLE: systemic lupus erythematosus

## 1 Introduction

Osteoarthritis (OA) is the most prevalent musculoskeletal disorder. OA induces joint pain and physical disability and increases the risk of complications, sleep disturbances, reduced capability for exercising, lifting, and walking and are less capable of working [1]. The etiology of OA is multifactorial and includes many factors such as age, sex, overweight, prior joint injury, medication, environmental factors, and genetics [[2], [3], [4]]. OA can cause progressive loss and destruction of cartilage, sclerosis of the subchondral bone, and inflammation of the synovium, which eventually lead to continuous arthritis. Symptomatic OA may also impair the ability to be physically active and to lose weight, which can lead to the vicious cycle of weight gain and continued physical inactivity and increased risk of associated conditions. Thus, treatment of OA is critical to maintaining physical ability and improving healthy life expectancy.

Traditionally, OA management includes nonpharmacological treatments such as exercise, weight loss, and patient education, and pharmacological treatments including anti-inflammatory medications and intra-articular injections. These treatments are effective for the early to intermediate phases of OA but are insufficient for progressive OA. Surgical options are considered for patients with progressive OA, but their effects are often temporary, and OA continues to progress [5]. In addition, patients with OA often have risk factors that can be contraindications for surgery, such as old age and other conditions. Thus, novel interventions to restore degraded cartilage or decelerate OA disease progression are required.

The use of adipose-derived stem/stromal cells (ADSCs) has recently been applied clinically for cartilage regeneration [6,7]. Since the discovery and characterization of multipotent stem cells (MSCs) in adipose tissue by Zuk et al. [8], ADSCs have been identified as a source of MSCs. MSCs are generally nontumorigenic and can be isolated from many sites, including adipose tissue, bone marrow, umbilical cord, dental pulp, endometrium, synovial fluid, muscle, and peripheral blood [[9], [10], [11]]. ADSCs can be easily and safely harvested from abundant adipose depots with low risk to the patient [12]. These cells can secrete many kinds of cytokines and growth factors and recruit macrophages, which can play an immunosuppressive role after exposure to an inflammatory environment [13]. ADSCs are of interest for clinical use in cell-based therapy, and favorable outcomes have been reported for their use in the treatment of knee OA [14,15]. However, the mechanisms responsible for the therapeutic effects of ADSCs have been only partially investigated, and the long-term safety and side effects of this treatment remain unclear.

Recently, we found that some patients with OA who received ADSC treatment presented with severe arthritis and fever. Therefore, we have investigated the pathophysiology of this apparent severe arthritis that occurs after ADSC treatment using autoantibody screening of synovial fluid obtained from patients who received ADSC treatment.

## 2 Materials and methods

### 2.1 Patients and synovial fluid samples

Ethical approval for the study protocol was obtained by the institutional review board of Saitama Cooperative Hospital (approval number: 170304). Adult Japanese patients with OA of the knee who received ADSC treatment at Saitama Cooperative Hospital (Kawaguchi, Saitama, Japan) between June 2018 and October 2021 were enrolled. Clinical data were collected retrospectively from clinical chart review for each patient. Severe arthritis was defined as arthritis that is painful at rest, unresponsive to analgesics, and presents with difficulty walking. The experiments using remaining samples were performed under the approval and guidance of the Clinical Research Review Committee of Tokai University School of Medicine (approval number: 22R072) and review board of Saitama Cooperative Hospital.

### 2.2 ADSC treatment

Adipose tissue was collected by liposuction from the knees of patients with OA. ADSCs were isolated from the knee joints of patients with OA undergoing aesthetic or prosthetic surgery at Saitama Cooperative Hospital, following a well-established protocol [12]. With the patient under local anesthesia, 20 ml of abdominal fat tissue was collected in an operating room. The collected fat was kept in normal saline at 4 °C and mailed within 48 h to the cell processing center at CellSource Co., Ltd. (Shibuya, Tokyo, Japan). The entire 20 ml adipose tissue sample for each patient was cultured for 2 weeks. The medium was replaced every other day and, when they reached 70–80% confluence, the cells were detached, plated, expanded, and cryopreserved until the day of administration, as described previously [16]. For injection, the ADSCs were washed twice, and 15 million cells were diluted with 3 ml of saline and injected into the intra-articular space of the knee. Some patients received this injection two or three times at 6-month intervals.

### 2.3 Antibody (Ab) detection

Ab in synovial fluid was screened by immunoprecipitation (IPP) using radiolabeled HeLa cell extracts as previously described [17]. Briefly, 1 × 10^7^ HeLa cells were labeled with 9.25 MBq of [^35^S]-methionine (PerkinElmer, Waltham, MA, USA) in 10 ml of methionine-free minimal essential medium and incubated at 37 °C for 18 h. The labeled cells were washed three times with phosphate-buffered saline (137 mmol/l NaCl, 8.1 mmol/l Na_2_HPO_4_, 2.68 mmol/l KCl, 1.47 mmol/l KH_2_PO_4_) and sonicated in IPP buffer (10 mM Tris–HCl, 500 mM NaCl, 0.1% Nonidet P-40). The soluble supernatant in IPP buffer was collected by centrifugation at 10,000 g for 10 min. Two microliters of synovial fluid was bound to 2 mg of Protein A CL-4B Sepharose beads (GE Healthcare, Uppsala, Sweden) in IPP buffer for 2 h at room temperature under constant rotation. After washing four times with IPP buffer, IgG-coated Sepharose beads were mixed with the [^35^S]-methionine-labeled HeLa cell extracts for 2 h at 4 °C. After washing four times with IPP buffer, the IgG-coated beads were resuspended in sodium dodecyl sulfate (SDS) sample buffer and heated for 5 min at 95 °C. The supernatant was subjected to SDS-polyacrylamide gel electrophoresis (PAGE) and visualized by radiography.

### 2.4 Identification of the autoantigen recognized by the anti-15 kDa Ab

To identify the corresponding autoantigen, immunoprecipitated polypeptides from HeLa cells without [^35^S]-methionine labeling were visualized by silver staining. The corresponding silver-stained gel band at 15 kDa was excised and subjected to liquid chromatography coupled with time-of-flight MS and ion trap MS (LCMS-IT-TOF) analysis.

### 2.5 Immunoblotting

Immunoblotting to confirm the antigen identity was performed according to a modified procedure detailed previously [17]. In short, Recombinant Human Histone H2B protein (NBP3-07913, Novus Biologicals, Centennial, CO, USA) was electrophoretically separated by SDS-PAGE using a 4–15% Mini-PROTEAN® TGX™ gel (Bio-Rad, Hercules, CA, USA) and later transferred to a polyvinylidene fluoride (PVDF) membrane. After transfer, the PVDF membrane was stained with Ponceau S Staining Solution (BCL-PSS-01, Beacle, Inc., Sakyo, Kyoto, Japan). After confirming the transferred protein, the PVDF membrane was washed, and each lane was cut out and incubated with 5% nonfat dry milk in Tris-buffered saline with Tween 20 (TBST, pH8.0) for 1 h at room temperature. The blots were washed with TBST three times for 5 min each and then incubated overnight at 4 °C with primary Abs contained in the synovial fluid obtained from patients with OA, serum from healthy controls, commercial anti-histone H2B Ab (GTX129434, GeneTex, Irvine, CA, USA), and anti-histone-Ab-positive serum from a patient with systemic lupus erythematosus (SLE). The membranes were washed with TBST five times for 5 min each, incubated with the second Ab against human or rabbit IgG (anti-human IgG AP conjugate, S3821 and anti-rabbit IgG AP conjugate, S3731, Promega K·K., Tokyo, Japan). The blots were washed five times with TBST and developed with BCIP/NBT Color Development Substrate following the manufacturer's instructions (Promega S3771).

### 2.6 Enzyme-linked immunosorbent assay (ELISA) to measure anti-histone H2B Ab titer

Synovial fluid samples were frozen and used for the measurement of anti-histone H2B Ab titer by ELISA using a Human Histone H2A-H2B-DNA Autoantibody ELISA Kit (MyBioSource, MBS029740, San Diego, CA, USA). The ELISA plate was read at 450 nm, and the cutoff value as an indicator of a negative response to anti-histone H2A-H2B-DNA Ab was defined as the mean plus 0.15 of the absorbance of the negative controls according to the manufacturer's instructions.

### 2.7 Tandem mass spectrometry (MS/MS) and protein identification

Identification of the protein in the gel spots was performed using an LCMS-IT-TOF instrument (Shimadzu, Kyoto, Japan). The pieces of interest were excised from the gel, and in-gel digestion of proteins was performed. The gel pieces were washed with 30% acetonitrile in 50 mM NH_4_HCO_3_ followed by 60% acetonitrile in 50 mM NH_4_HCO_3_, and the samples were dried using a SpeedVac concentrator (Thermo Fisher Scientific, Waltham, MA, USA). For in-gel digestion of proteins, the samples were incubated with a trypsin solution at 37 °C overnight, and the extracted trypsinized peptides were eluted stepwise with 30%, 100%, and 0% of acetonitrile in 50 mM NH_4_HCO_3_. The samples were dried and then dissolved in 50 mM NH_4_HCO_3_. Tryptic peptides were separated by reversed-phase liquid chromatography/MS using a Prominence nanoLC instrument (Shimadzu, Kyoto, Japan) for analytical separation on a PicoFrit^TM^BetaBasic C18 column (100 mm × 75 μm; New Objective, Woburn, MA, USA). MS was performed on an LCMS-IT-TOF instrument with argon gas for ion cooling and collision-induced dissociation experiments. MS/MS data were obtained in a data-dependent manner. The Mascot search engine (Matrix Science, Boston, MA, USA) was used for protein database searching. Proteins with a statistically significant MASCOT/Mowse score (>28), indicating identity or extensive homology (*P* < 0.05), were considered for identification.

## 3 Results

### 3.1 Severe arthritis induced by ADSC injection

A total of 113 patients with knee OA received ADSC treatment; 75% (85/113) had received ADSC injection twice or more at 6-month intervals. No obvious abnormalities were observed in any patient after their first treatment. However, surprisingly, 53% (45/85) of patients who received their second ADSC injection showed joint swelling, severe pain, and redness. Fever was noted along with severe arthritis in 11% (9/85) of patients who received a second ADSC injection. Considering the possibility that multiple ADSC treatments had induced severe arthritis, we investigated the pathogenesis by screening for autoantibodies in synovial fluid from the patients who showed severe arthritis.

### 3.2 Screening for anti-15 kDa Abs

**Fig. 1:**
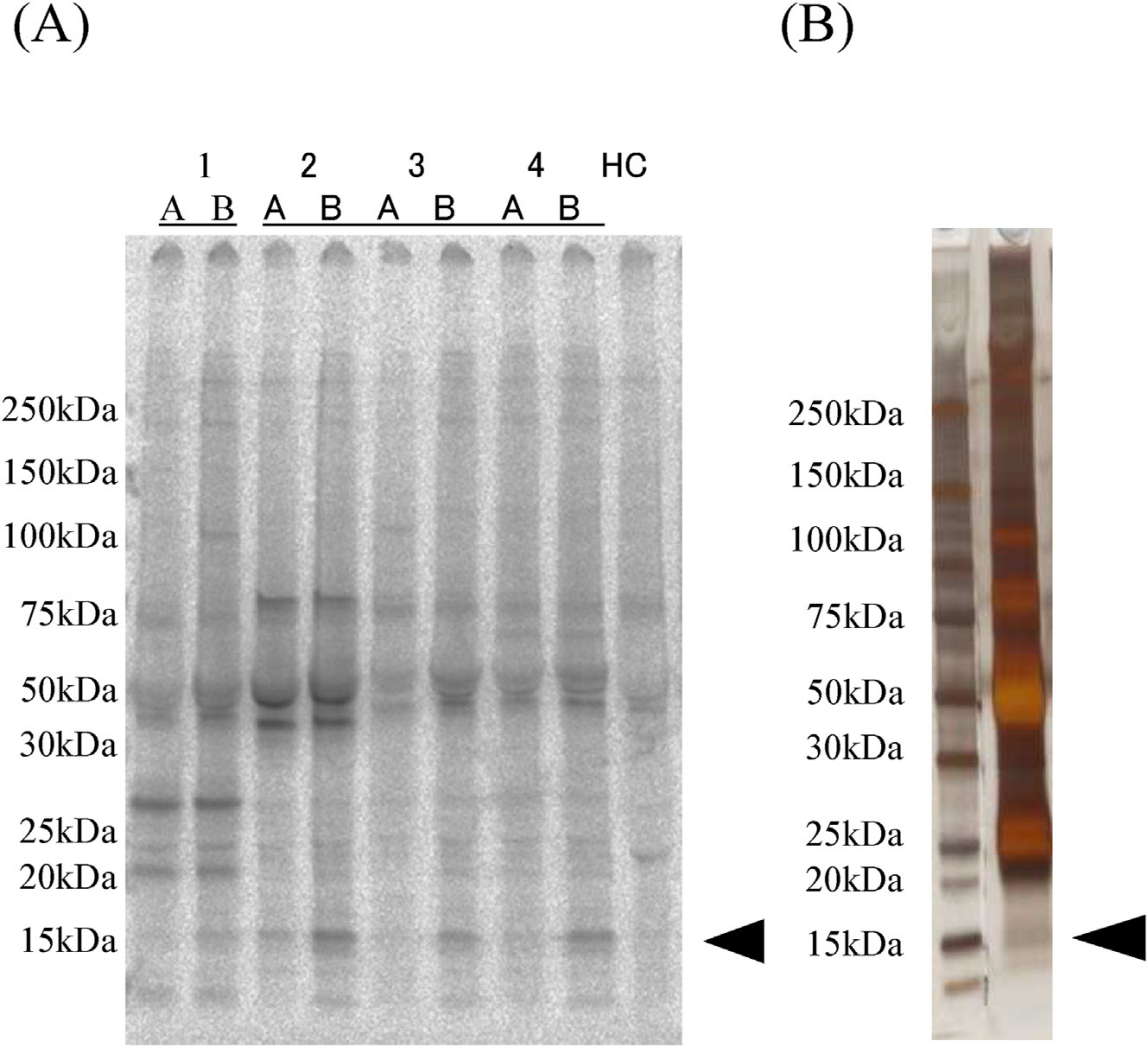
IPP of polypeptides with patient synovial fluid samples and silver staining. (A) Synovial fluid from patients with OA contained the anti-15 kDa antibody. Using [^35^S] methionine-labeled HeLa cell extracts, antigens were precipitated with the synovial fluid from patients with OA (1–4) and serum from healthy control. HC; healthy control, A; before treatment, B; post second injection. (B) HeLa cell extracts reacted with Patient No.4B synovial fluid were fractionated on SDS–polyacrylamide gel and visualized by silver staining.

Synovial fluid was available both before and after the second or third injections for eight of the 45 patients (18%) who showed severe arthritis after multiple ADSC injections. We used IPP with [^35^S]-methionine-labeled HeLa cells to screen for autoantibodies in synovial fluid obtained from these patients (Fig. 1). A common 15 kDa polypeptide was immunoprecipitated from the synovial fluid of 62% (8/13) of the samples analyzed (Fig. 1A, black arrow). Interestingly, the 15 kDa polypeptide was not precipitated from the synovial fluid from any patient after the first injection but was immunoprecipitated from the synovial fluid obtained from patients after the second and third injections (Fig. 1A, pre: before treatment, and post: after the second or third injection). Interestingly, none of the synovial fluid samples obtained from any patient after the first injection immunoprecipitated the 15 kDa polypeptide, but those obtained from patients who showed severe arthritis after the second and third injections did (Fig. 1A, pre: before treatment, and post: after the second or third injection). It was not detected in samples from patients without severe arthritis.

### 3.3 Identification of the autoantigen recognized by the anti-15 kDa Ab

**Fig. 2:**
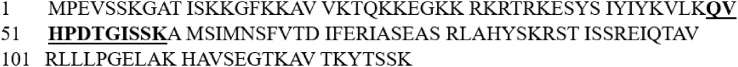
MASCOT search results. The silver-stained band corresponding to 15 kDa was excised and digested with trypsin. The peptide mixture was analyzed in duplicate with MALDI-TOF MS. MALDI-TOF spectra of peptide ion masses from the protein digest were used to predict the protein sequence. Mascot was used to compare the observed spectra with a database of known proteins and determine the most likely matches. The results showed histone H2B as the candidate protein corresponding to the immunoprecipitated autoantigen. Protein sequence coverage was 8%.

Next, to identify the immunoprecipitated common 15 kDa protein, synovial fluid that had immunoprecipitated the 15 kDa protein was coupled with Protein A CL-4B Sepharose beads and incubated with HeLa cell extracts without [^35^S]-methionine labeling. The polypeptides were then fractionated by SDS-PAGE and visualized by silver staining (Fig. 1B). The band corresponding to 15 kDa was cut out and subjected to LCMS-IT-TOF analysis, and this analysis suggested histone H2B as the candidate corresponding protein (Fig. 2).

### 3.4 Confirmation of histone H2B as the corresponding autoantigen to anti-15 kDa Ab

**Fig. 3:**
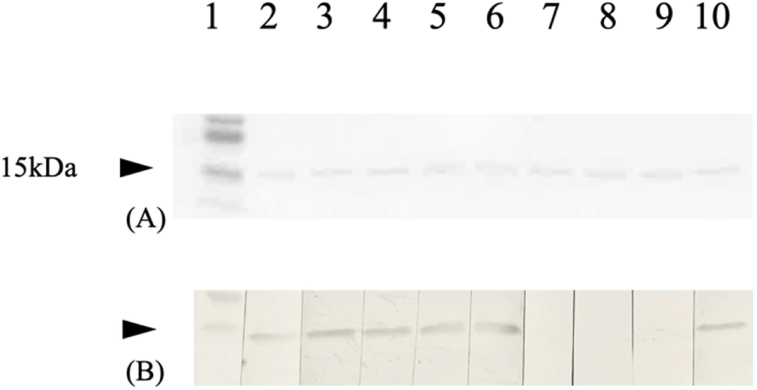
Immunoblot analysis of histone H2B protein as the corresponding autoantigen. (A) Ponceau S-stained PVDF membrane. Recombinant human histone H2B protein was separated electrophoretically by SDS-PAGE using a 4–15% Mini-PROTEAN® TGXTM gel, transferred to a PVDF membrane, and stained with Ponceau S to confirm protein transfer. (B) After cutting the each lane, the membranes were reacted with the anti-15 kDa-positive synovial fluid, anti-15 kDa-negative synovial fluid, commercial anti-histone H2B antibody, and anti-histone H2B-positive serum from a patient with SLE. Lane 1: molecular weight marker; lane 2: commercial anti-histone H2B antibody; lanes 3–6: anti-15 kDa-positive synovial fluid; lanes 7–9: anti-15 kDa-negative synovial fluid; and lane 10: anti-histone H2B-Ab-positive serum from a patient with SLE.

To confirm that histone H2B was recognized by the anti-15 kDa Ab, immunoblotting using recombinant human histone H2B protein was performed. Histone H2B protein on the PVDF membranes reacted with the commercial anti-histone H2B Ab, synovial fluid samples that immunoprecipitated the common 15 kDa protein, and anti-histone H2B-positive serum from a patient with SLE, whereas samples without the anti-15 kDa Ab did not (Fig. 3). This confirmed that histone H2B was the autoantigen corresponding to the anti-15 kDa Ab.

### 3.5 Anti-histone H2B Ab titer before and after ADSC treatment

To identify when anti-histone H2B Ab appeared and to measure changes in Ab titers, synovial samples that were available both before and after ADSC treatment were analyzed by ELISA (Table 1). Interestingly, all of these samples were negative for anti-histone H2B Ab before treatment and were newly positive only after treatment. These results suggest that anti-histone H2B Ab did not exist in the synovial fluid before ADSC treatment but appeared only after multiple ADSC treatment.

**Table 1:** Patients who experienced knee pain and presence of anti-human histone H2A-H2B-DNA autoantibody (HHDNA) in their synovial fluid before and after treatment.

Patient number	Age (years)	Sex	Num-ber of ADSC treat-ments	KL grade	Knee joint	Presence of anti-HHDNA in synovial fluid before and after treatment		Volume of synovial fluid aspirate (mL)		Appea-rance of synovial fluid aspirate	Duration of swelling, heat, and pain after ADSC injection (days)
						Before	After	Before	After^e^		
1	83	F	2	4	R	–	+	5.8	12.0	PY^d^	5
				4	L	–	+	1.0	2.0	PY	
2	86	F	2	4	R	–	+	8.0	8.0	PY	7
				4	L	–	+	6.0	6.0	PY	
3	63	M	2	3	R	–	+	15.0	14.0	PY	1
4	56	F	2	4	R	–	+	8.5	1.0	PY	4
				2	L	–	+	17.0	14.0	PY	
5^a^	76	F	3	3	L	–	+	7.2	9.2	PY	30
6	52	F	2	4	R	–	+	12.0	35.0	PY	2
				4	R		+		2.0	PY	
7^b^	64	F	2	2	L	–	+	7.0	6.8	PY	1
8^c^	66	F	3	3	R	–	+	4.8	12.2	PY	10

a Patient 5 received ADSC injection into the right knee in the first treatment, into both knees in the second treatment, and into the left knee in the third treatment. Both knees showed severe pain and swelling after the second ADSC injection.

b Patient 7 received ADSC injection into the hip in the first treatment.

c Patient 8 experienced knee pain after the second and third ADSC treatment. Pain was present for 10 days after the second, and severe pain for 1 week after the third.

d PY: Pale yellow plasma-like.

e Synovial fluid was aspirated 1 week after treatment for patient 1-4, 2 weeks for patient 5, 1 and 4 weeks for patient 6 and 4 weeks for patient 7 and 8.

## 4 Discussion

This study reports some novel findings regarding ADSC treatment in patients with knee OA. First, we found that severe knee arthritis was induced in a high percentage of patients after multiple ADSC injections. Second, IPP detected anti-histone H2B Ab in synovial fluids from the patients who showed severe knee arthritis after treatment. Third, anti-histone H2B Ab appeared to be newly induced after multiple ADSC treatment.

OA is the most common form of arthritis and is treated in many ways including intra-articular injection. However, OA is generally progressive and leads to irreversible destruction of cartilage, which is a major cause of disability. Although the exact pathogenesis of knee OA is unclear, continuous inflammation throughout the entire joint structure leads to the degradation of articular cartilage, formation of subchondral bone, and mild synovitis [18]. Synovial tissues from people with OA demonstrate the infiltration of CD4^+^ and CD68^+^ cells, formation of blood vessels, and expression of vascular endothelial growth factor and intracellular adhesion molecule 1 [19]. The expression of cytokines such as tumor necrosis factor α, interleukin 1 (IL-1), IL-6, and IL-17, which activate CD68^+^ macrophages, is also highly upregulated in joints with OA [[18], [19], [20], [21], [22], [23], [24]].

The anti-inflammatory effects of ADSC treatment have been reported recently. For example, the intra-articular deposition of ADSCs inhibited thickening and activation of the synovial lining layer and protected against joint destruction in an experimental OA model involving synovial activation [25]. In addition, the process of acquisition of ADSCs from adipose tissue allows for the development of anti-inflammatory properties and the suppression of inflammatory agents produced by fat [26]. Thus, ADSC treatment for OA is considered a potent and safe tool for managing inflammation and protecting against the joint destruction caused by OA. However, in our study, 53% of the patients with knee OA who were treated with ADSC injection showed symptoms of severe knee arthritis, especially after their second or third injection, and some patients developed fever. Although previous reports have indicated the utility of ADSC treatment for patients with OA, few studies have reported on the side effects of this procedure, and its long-term safety remains unclear.

We found the identification of anti-Histone H2B Ab production with severe knee arthritis after ADSC injection, especially after multiple injections. Of note is the high frequency of anti-histone H2B Ab detected in the synovial fluid from those patients who showed symptoms of severe knee arthritis. The corresponding autoantigen histone is a protein component found in the nucleosome and consists of five subunits: H1 (21 kDa), H2A (14.5 kDa), H2B (13.7 kDa), H3 (15.3 kDa), and H4 (11.3 kDa). Each nucleosome contains core histones that form a spool that wraps 145–147 bp of DNA [27]. Autoantibodies to histones were first reported in the serum from patients with SLE and were later detected in the serum from patients with drug-induced lupus erythematosus (DILE) [[28], [29], [30]]. It was estimated that 24–95% of patients with SLE and 67–100% of those with DILE are positive for anti-histone Ab [31]. Among the five subunits of histone H2B, histones H1 and H2B are prominent autoantigens that induce the production of anti-histone Abs [32], H2B is the corresponding consistent with the results of our study.

Many drugs can induce DILE symptoms such as arthritis, fever, muscle pain, or skin lesions [30]. Procainamide and hydralazine are known triggers of lupus-like symptoms and anti-histone H2B autoimmunity. These symptoms are similar to those associated with SLE, although the autoantibodies differ between DILE and SLE. For example, anti-Sm and anti-RNP Abs are often detected in serum from patients with SLE, whereas both are rare in serum from patients with DILE [30]. Although the pathogenic mechanisms remain unclear, DILE is temporally related to continuous exposure to some medications and resolves after discontinuation of the medication. Arthritis is the most frequent, but often the only, clinical symptom and occurs in 90% of patients with DILE. In our study, none of synovial fluid samples from the patients who showed severe arthritis with anti-histone H2B Ab was positive for anti-Sm or RNP Ab by IPP screening. Moreover, none of the cases of severe knee arthritis was induced by a single injection, and symptoms appeared only after the second injection; that is, anti-histone H2B Ab appeared to be newly induced only after multiple ADSC treatment. Given the clinical course and the appearance of anti-histone H2B Ab, severe knee arthritis induced by multiple ADSC injections may be similar to DILE and may reflect an ADSC-induced autoimmune response.

Although our present report differs from conventional reports regarding safety, the present report is not immediately intended to deny or cast doubt on the efficacy or safety of ADSC treatment. One article indicates that 10% of patients who received single ADSC injection had pain and swelling for 4 weeks following ADSC therapy, and due to the observed impact on their usual activities of daily living, it was categorized as a severe adverse event. Nevertheless, none of those in a group with double ADSC injections were detected as a severe adverse event [33]. Sekiya et al. reported that multiple MSC injections for patients with progressive OA suppressed cartilage loss without a severe adverse event [34]. Another article indicated that 50%–60% of those in an MSC treatment group experienced inflammation and swelling after the injection procedure [35]. However, the same symptoms were also observed in a control group. These reports support the safety and efficacy of ADSCs and the possibility of adverse events being due to the course of administration rather than ADSCs themselves. Because anti-histone H2B Ab production was observed along with severe arthritis, which suggests some type of immune response was induced, further investigation is required to fully elucidate this point.

## 5 Limitations

This study has three main limitations. First, synovial fluid was not collected at the time of each ADSC treatment because we could not predict that severe knee arthritis would occur in such a high percentage of patients after the second injection. Second, control group was not set since ADSC treatment was in clinical practice. Third, the patients were not treated with glucocorticoids, which is commonly used for patients with DILE, and we were unable to confirm whether the clinical course and pathophysiology of the symptoms in these patients were fully compatible with those of DILE. Future prospective studies are needed to clarify the pathophysiology of severe arthritis induced by ADSC treatment.

## 6 Conclusions

We investigated the pathophysiology of severe arthritis that appeared after ADSC treatment for OA of the knee. Anti-histone H2B Ab was detected in synovial fluid samples from those patients who exhibited severe arthritis. Anti-histone H2B Ab production was induced after the second ADSC treatment. These findings provide information that may be useful in the development of improved treatments for OA involving ADSCs.

## Authors’ contributions

Conception and design: YH, AK, ET, KN, SS, and MS; data acquisition: AK, ET, KN, and MS; data analysis and interpretation: YH, AK, ET, KN, and MS; drafting of the manuscript: YH, ET, and MS; and revision of the manuscript for important intellectual content: YH, ET, MW and MS. All authors have read and approved the final manuscript.

## Declarations of competing interest

The authors declare that they have no known competing financial interests or personal relationships that could have appeared to influence the work reported in this paper.
